# Methionine restriction alters bone morphology and affects osteoblast differentiation

**DOI:** 10.1016/j.bonr.2016.02.002

**Published:** 2016-02-11

**Authors:** Amadou Ouattara, Diana Cooke, Raj Gopalakrishnan, Tsang-hai Huang, Gene P. Ables

**Affiliations:** a Orentreich Foundation for the Advancement of Science, Inc, 855 Route 301, Cold Spring, NY 10516, USA; b School of Dentistry, University of Minnesota, Minneapolis, MN 55455, USA; c Institute of Physical Education, Health and Leisure Studies, National Cheng Kung University, Tainan City, Taiwan

**Keywords:** BMC, bone mineral content, BS, bone surface, BV, bone volume, CF, control-fed, Conn.Dn., connectivity density, CTX-1, C-terminal telopeptide of type 1 collagen, FGF21, fibroblast growth factor-21, HFD, high-fat diet, HHCy, hyperhomocysteinemia, IDI, indentation depth increase, IGF-1, insulin-like growth factor-1, *I*_*max*_, maximal MOI, *I*_*min*_, minimal MOI, LPD, low protein diet, MOI, moment of inertia, MR, methionine restriction, OC, osteocalcin, OPG, osteoprotegerin, P1NP, N-terminal propeptide of type 1 procollagen, pMOI, polar MOI, RANKL, receptor activator for nuclear factor κB ligand, SMI, structure model index, Tb.N, trabecular number, Tb.Sp, trabecular separation, Tb.Th, trabecular thickness, TV, total volume, vBMD, volumetric bone mass density, μCT, micro-computed tomography, Methionine restriction, Aged mice, Micro-computed tomography, Nanoindentation, MC3T3-E1 subclone 4

## Abstract

Methionine restriction (MR) extends the lifespan of a wide variety of species, including rodents, drosophila, nematodes, and yeasts. MR has also been demonstrated to affect the overall growth of mice and rats. The objective of this study was to evaluate the effect of MR on bone structure in young and aged male and female C57BL/6J mice. This study indicated that MR affected the growth rates of males and young females, but not aged females. MR reduced volumetric bone mass density (vBMD) and bone mineral content (BMC), while bone microarchitecture parameters were decreased in males and young females, but not in aged females compared to control-fed (CF) mice. However, when adjusted for bodyweight, the effect of MR in reducing vBMD, BMC and microarchitecture measurements was either attenuated or reversed suggesting that the smaller bones in MR mice is appropriate for its body size. In addition, CF and MR mice had similar intrinsic strength properties as measured by nanoindentation. Plasma biomarkers suggested that the low bone mass in MR mice could be due to increased collagen degradation, which may be influenced by leptin, IGF-1, adiponectin and FGF21 hormone levels. Mouse preosteoblast cell line cultured under low sulfur amino acid growth media attenuated gene expression levels of *Col1al*, *Runx2*, *Bglap*, *Alpl* and *Spp1* suggesting delayed collagen formation and bone differentiation. Collectively, our studies revealed that MR altered bone morphology which could be mediated by delays in osteoblast differentiation.

## Highlights

• MR affected the growth rates of males and young females, but not aged females.
• CF and MR mice had similar intrinsic strength properties.
• Low methionine media attenuated bone differentiation genes in MC3T3-E1 preosteoblast cells.
• The lower bone mass in MR mice is appropriate for its smaller body size.

## 1 Introduction

Methionine restriction (MR) extends the lifespan of a wide variety of species, including rodents, drosophila, nematodes, and yeasts (Brown-Borg et al., 2014, Cabreiro et al., 2013, Johnson and Johnson, 2014, Lee et al., 2014, Miller et al., 2005, Orentreich et al., 1993, Richie et al., 1994, Ruckenstuhl et al., 2014). Lifespan extension by MR in rodents could be due to several factors, including: 1) delays in age-related diseases, such as obesity and diabetes (Ables et al., 2012, Perrone et al., 2008, Plaisance et al., 2011, Stone et al., 2014); 2) decreased mitochondrial oxidative stress (Caro et al., 2008, Sanchez-Roman and Barja, 2013); and 3) reduced risk for cancer progression (Komninou et al., 2006, Lu et al., 2002, Sinha et al., 2014). In addition to the beneficial effects of MR, this diet also reduces the body size of mice and rats (Ables et al., 2012, Huang et al., 2014).

To our knowledge, there are only two previous studies that investigated the effects of MR in bones of young growing male mice and rats (Ables et al., 2012, Sinha et al., 2014). We previously reported that mice provided a high-fat methionine-restricted diet (HFD-MR) exhibited growth restriction compared to their HFD-CF (control-fed) counterparts, which could be due to increased collagen degradation (Ables et al., 2012). The HFD-MR mice had smaller femurs with reduced bone mass density (BMD) and bone mineral content (BMC) compared to HFD-CF mice (Ables et al., 2012). Huang et al. reported that MR rats were smaller; had reduced bone mass compared to CF rats, as determined by micro-computed tomography (μCT); and had decreased extrinsic strength, as measured by a 3-point bending test (Huang et al., 2014). However, bones from MR rats had higher intrinsic biomaterial strength and toughness compared to CF rats (Huang et al., 2014). These studies suggest that MR affects overall bone development in rodents.

A salient characteristic of MR is its ability to induce hyperhomocysteinemia (HHcy) in rodents due to decreased cystathionine β-synthase (CBS) activity (Ables et al., 2015, Elshorbagy et al., 2010). Interestingly, HHCy, an independent risk factor for cardiovascular disease, did not alter cardiac function in MR mice (Ables et al., 2015). In addition, Tyagi et al. reported that HHcy in mice reduced bone mass and affected bone remodeling due to altered bone blood flow (Tyagi et al., 2011). Furthermore, Vijayan et al. reported that HHCy induced alterations in osteoprotegerin/RANKL ratio suggesting increased osteoclast activity which could lead to bone loss (Vijayan et al., 2013). Our current studies, however, focused on the effects of MR in bone metabolism which could be indirectly affected by HHCy.

The potential to translate the benefits of MR into the clinical setting due to its favorable effects with regard to the prevention of diabetes, obesity, and cancer is increasing (Ables et al., 2012, Perrone et al., 2008, Plaisance et al., 2011, Komninou et al., 2006, Sinha et al., 2014, Lu et al., 2003).

To contribute to the overall understanding of MR, we conducted a study on its effects on bone growth and development. Our current study focused on the bones of young and aged mice. Importantly, our studies addressed whether gender is a factor in the effects of MR, which has not been fully explored in the field of bone biology. Finally, we identified a potential molecular mechanism of MR in bones using MC3T3-E1 preosteoblast cell line.

## 2 Materials and methods

### 2.1 Animal care

All of the experiments were approved by the Institutional Animal Care and Use Committee of the Orentreich Foundation for the Advancement of Science, Inc. (Permit Number: 0511MB). Male and female C57BL/6J (Stock #000664) mice were purchased from Jackson Laboratories (Bar Harbor, ME, USA) and individually housed in a conventional animal facility maintained at 20 ± 2 °C and a 50 ± 10% relative humidity with a 12 h light: 12 h dark photoperiod. Young males and females were 8 weeks old at the initiation of the experiments and 20 weeks old upon termination. Aged male and female mice (retired breeders from the Jackson Laboratories) were 9 months old at the initiation of the experiments and 12 months old upon termination. Food and water were provided ad libitum. The diet ingredients and feeding protocol have been described previously (Ables et al., 2012, 2015). Briefly, upon arrival, the mice were acclimatized for one week and fed Purina Lab Chow #5001 (St. Louis, MO, USA). Afterwards, they were weight matched and separated into either CF (0.84% methionine w/w) or MR (0.12% methionine w/w) diets consisting of 14% kcal protein, 76% kcal carbohydrate, and 10% kcal fat (Research Diets, New Brunswick, NJ, USA) for 12 weeks. The diet compositions are shown in Supplementary Table 1. Body weight and food consumption were monitored twice weekly. On the day of sacrifice, animals were fasted for 4 h at the beginning of the light cycle to establish a physiological baseline. Mice were sacrificed by CO_2_ asphyxiation. Blood was collected from the retro-orbital plexus, and plasma was collected, flash frozen, and stored at − 80 °C until analyzed.

### 2.2 Animal measurements and bone sample preparation

Under light isoflurane anesthesia, length measurements were made from the tip of the nose to the base of the tail of each mouse every 4 weeks for young mice and every 2 weeks for aged mice. After sacrifice, the bones were separated from the soft tissue and processed as described previously (Ables et al., 2012, Huang et al., 2014). Briefly, the tibiae were stored in 70% ethanol for μCT scanning. The femur length was measured using a caliper ruler from the head of the femur to the distal condyle. The bones were then cleaned of soft tissue, wrapped in gauze, immersed in PBS (pH 7.4), and stored in aluminum foil at − 80 °C for nanoindentation test. Bones were collected according to different tests, as described below.

### 2.3 Micro-computed tomography (μCT) analysis

Bone histomorphometry was conducted as described previously (Huang et al., 2014). Briefly, tibiae that were subjected to μCT scanning (SkyScan 1176, SkyScan, Belgium) using the following parameters: Al 0.5-mm filter, 48 kV, 200 μA, 1° rotation step per picture with 2600 ms exposure time, and 9 μm pixel size. Cross-section images (8-bit BMP file) were reconstructed using NRecon (version 1.6.9.4, SkyScan, Belgium) with the following parameters: dynamic range = 0–0.13, smoothing = 2, ring artifact correction = 6, and beam hardening correction (%) = 22. Various densitometry and histomorphometry analyses were performed using CT-Analyzer (version 1.12.0.0; SkyScan) with the gray threshold consistently selected over a range of 50–255. Volumetric bone mass density (vBMD, g/cm^3^) and bone mineral content (BMC, mg) measurements were conducted on the whole tibiae, midshaft cortical bones (transverse slices of 1 mm in thickness), and secondary spongiosa of each tibia (transverse slices between 0.5 and 2.5 mm below the lowest point of the growth plate at the distal metaphysis without cortical bone). The following were measured in secondary spongiosa: histomorphometric indices of bone volume (BV, mm^3^) and the BV to total volume (TV) ratio (BV/TV, %), bone surface (BS, mm^2^), BS to BV ratio (BS/BV, 1/mm), trabecular thickness (Tb.Th, mm), trabecular number (Tb.N, 1/mm), trabecular separation (Tb.Sp, mm), connectivity density (Conn.Dn., 1/mm^3^), and structure model index (SMI). In addition, a transverse-CT slice was acquired to assess cross-sectional parameters, including the bone area (mm^2^) and 3 indices of cross-sectional moment of inertia (MOI): polar MOI (pMOI), maximal MOI (*I*_*max*_), and minimal MOI (*I*_*min*_).

### 2.4 Bone material testing by nanoindentation

The material level intrinsic properties were assessed using a nanoindentation system (MTS/Agilent XP, Santa Clara, CA, USA) consisting a 60 ± 5° diamond conical indenter with a radius of 2 μm at the tip (Dubois-Ferriere et al., 2014). In brief, femurs were thawed at room temperature, glued to an aluminum stage, and moistened with PBS (pH 7.4) solution for the indentation test. The test was performed on the posterior cortical bone surface at the midshaft of the femur. For each indentation location, two identical trials were performed using the following protocol: ultimate load of 100 milli-Newtons (mN), loading/unloading rate of 1 mN/s, and ultimate-load holding time of 30 s. After 5 min of recovery for the viscoelastic property, a second identical test was conducted on the same location. The indentation hardness (H) and modulus (*E*) were calculated using the following equations:

H=P/A

$$E=\frac{1}{2}∙\frac{\sqrt{\pi }}{\sqrt{A}}∙\frac{\mathrm{dP}}{\mathrm{dh}}$$

where *H* is hardness, *P* is the indentation load, *A* is the projected contact area, *E* is the indentation modulus, and $\frac{\mathrm{dP}}{\mathrm{dh}}$ is the maximal slope of the unloading curve. In addition, the distance between two ultimate load depths and two indentations on the same location was measured as the indentation depth increase (IDI, nm) (Hansma et al., 2008). For each femur sample, the repetitive indentation trial was conducted in two locations at a distance of 0.5 mm from each other.

### 2.5 Blood biochemical tests

ELISA kits were used to detect the N-terminal propeptide of type 1 procollagen (P1NP), C-terminal telopeptide of type 1 collagen (CTX-1) (Immunodiagnostic Systems, Fountain Hills, AZ), receptor activator for nuclear factor κB ligand (RANKL), leptin, insulin-like growth factor-1 (IGF-1), adiponectin (R&D Systems, Minneapolis, MN, USA); and fibroblast growth factor-21 (FGF-21, Millipore Corp., Billerica, MA, USA). Multiplex analysis was conducted using a Luminex 200 system at the Human Immune Monitoring Core at Mount Sinai Icahn School of Medicine (New York, NY) using the metabolites for osteoprotegerin (OPG) and osteocalcin (OC, MBNMAG-41K, Millipore Corp.).

### 2.6 Cell culture experiments

Mouse preosteoblast cell line MC3T3-E1 subclone 4 derived from murine calvaria was purchased from the American Type Culture Collection (CRL-2593, ATCC, Manassas, VA). Cells were cultured in α-modified Eagle's medium (α-MEM) containing 10% fetal bovine serum (FBS) (ATCC) under 37 °C in a humidified atmosphere of 5% CO_2_. Cells were passaged every 3 days using Trypsin-EDTA (30–2101, ATCC). For experiments, low passage cells were plated at a density of 5 × 10^5^/cm^2^ for 24 h until 80% confluent; cells were washed once with PBS solution and experimental culture media was added. To limit other amino acids in the experimental culture media, dialyzed FBS was used, as described previously (Ramalingam et al., 2010, Skrovanek et al., 2007). To differentiate cells into osteoblasts, 50 μg/ml ascorbic acid (Sigma) and 10 mM β-glycerophosphate (Sigma), were added to the culture media, as described previously (Wang et al., 1999, Xiao et al., 1997). For control media (CF), α-MEM (A10490 Thermo Fisher, Grand Island, NY) was used as complete media containing 100 mg/L cysteine, 31 mg/L cystine, and 15 mg/L methionine supplemented with 10% dialyzed FBS (Thermo Fisher). To mimic the MR diet in mice, sulfur amino acid restricted (SAAR) media was created from complete media diluted with custom α-MEM without cysteine, cystine, and methionine (Thermo Fisher). The final concentration of sulfur amino acids in the SAAR media was cysteine 20 mg/L, cystine 6.2 mg/L, and methionine 3 mg/L. Fresh media was added to the cells every 3 days. When cells were cultured in low methionine media in the absence of cysteine and cystine, a low rate of survival was observed (data not shown).

### 2.7 Gene expression analysis

For gene expression analysis in cells, Trizol (LifeTech) was added to each well of a 6-well cell culture plate following 2 washes of PBS at 24 h and 6 days after plating. Isolation of RNA from whole bones was conducted as described previously (Carter et al., 2012). Briefly, ice-cold Trizol was added to frozen whole bones and homogenized using Polytron (Kinematica, Bohemia, NY). Qiagen RNA isolation kits (Qiagen, Valencia, CA) were used to purify RNA from cells and bones. cDNA was prepared as described previously (Ables et al., 2012) and TaqMan quantitative PCR was conducted using primers for Alkaline Phosphatase (*Alpl*, Mm00475834_m1), Bone gamma carboxyglutamate or osteocalcin (*Bglap*, Mm03413826_mH), Collagen Type 1, alpha 1 (*Col1a1*, Mm00801666_g1), Collagen Type 2, alpha 1 (*Col2a1*, Mm01309565_m1), Runt-related transcription factor 2 (*Runx2*, Mm00501584_m1), and Secreted Phosphoprotein 1 or Osteopontin (*Spp1*, Mm00436767_m1). Gene expression was assessed by the comparative CT (ΔΔCT) method with β-actin as the reference gene; fold change was based on 24 h CF treated cells.

### 2.8 Statistical analyses

Data are presented as the mean ± standard deviation (SD). Comparisons between the two groups were conducted using ANOVA with a Bonferroni post hoc test for time course studies or Student's unpaired *t*-tests for endpoint analyses. All analyses were performed using Prism 6 (GraphPad Software, La Jolla, CA, USA).

## 3 Results

### 3.1 Variable effects of MR on growth in young and aged male and female mice

**Fig. 1:**
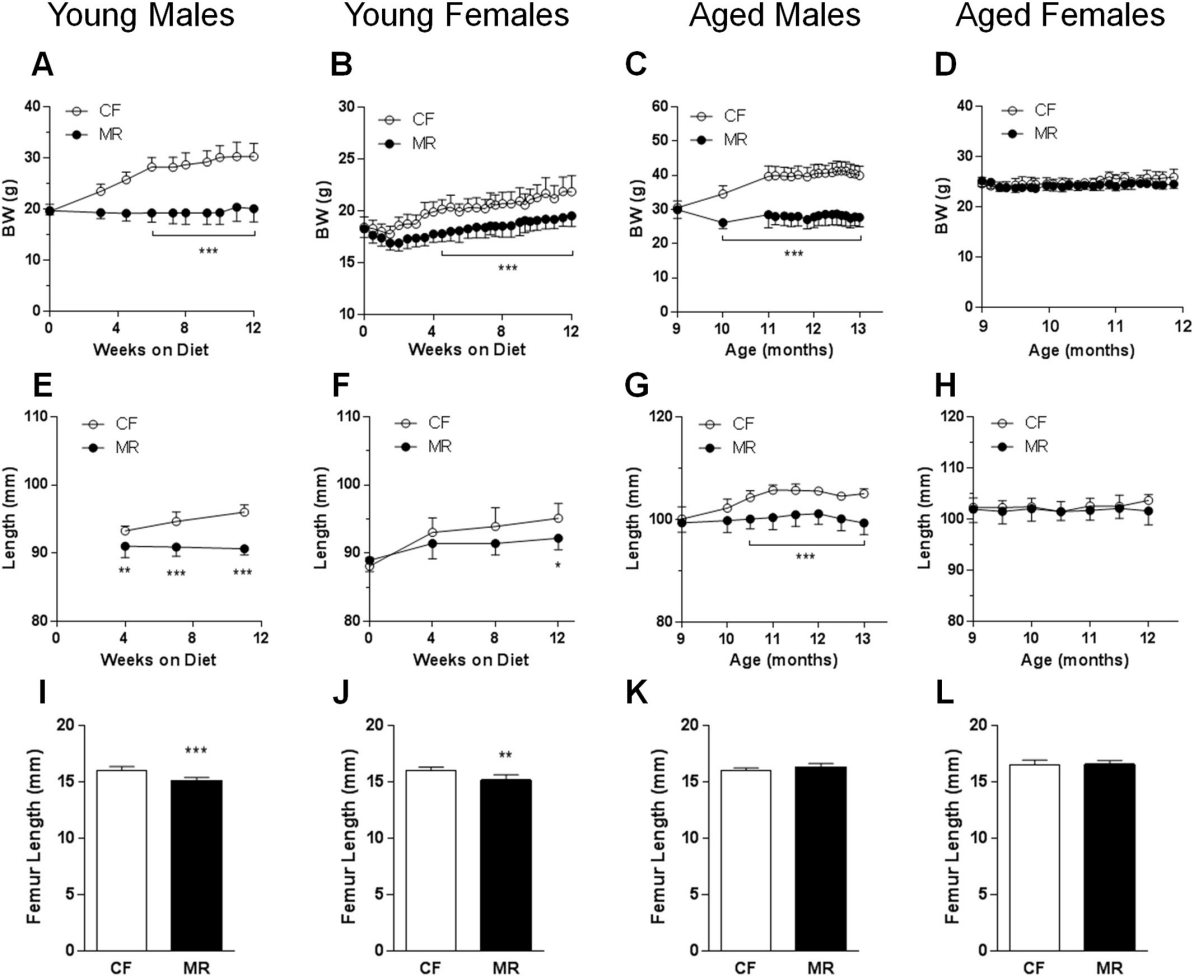
MR altered growth and body size in young and aged male and female mice. Body weights were measured during the course of the study for young males and female (A and B) and aged male and female mice (C and D) on CF (open circles) and MR (black dots) diets. Body length measurements from the tip of the nose to the base of the tail were measured in young male and female (E and F) and aged male and female (G and H) mice. Femur lengths were measured at the end of the diet studies from young male and female (I and J) and aged male and female (K and L) mice on CF (white bars) or MR (black bars). Statistical analysis was conducted using 2-ANOVA with Bonferroni post hoc tests for time-course studies or Student's unpaired *t*-test for endpoint comparisons between 2 groups (*n* = 7–8/group, *P < 0.05, **P < 0.01, ***P < 0.001).

To determine whether the effects of the MR diet on mouse body mass are age- and/or gender-related, we conducted studies in young and aged male and female mice that were fed CF and MR diets for 12 weeks. Our present data are consistent with our previous studies (Ables et al., 2012, 2015) in which young male MR mice had lower body weights after 12 weeks on diet (Fig. 1A*, P* < 0.001). In addition, young MR female and aged MR male mice had lower body weights than their CF counterparts (Fig. 1B and C, *P* < 0.001). On the other hand, aged female CF and MR mice maintained similar body weights for the duration of the study (Fig. 1D). Body length measurements revealed that young and aged male and young female MR mice were smaller than their CF counterparts (Fig. 1E–G, *P* < 0.05). Aged CF and MR female mice had similar body lengths for the duration of the study (Fig. 1H). The femur lengths of young male and female MR mice were shorter than their CF counterparts (Fig. 1I and J, *P* < 0.01), while the femur lengths of aged male and female CF and MR were similar (Fig. 1K and L). Our previous studies showed increased food consumption per gram body weight in young male MR mice (Ables et al., 2012, 2015), and we observed similar increases in food consumption in young MR female and aged MR male and female mice (Supplementary Fig. 1, *P* < 0.001).

### 3.2 The MR diet altered volumetric bone mass density (vBMD) and bone mineral content (BMC) in mice

**Fig. 2:**
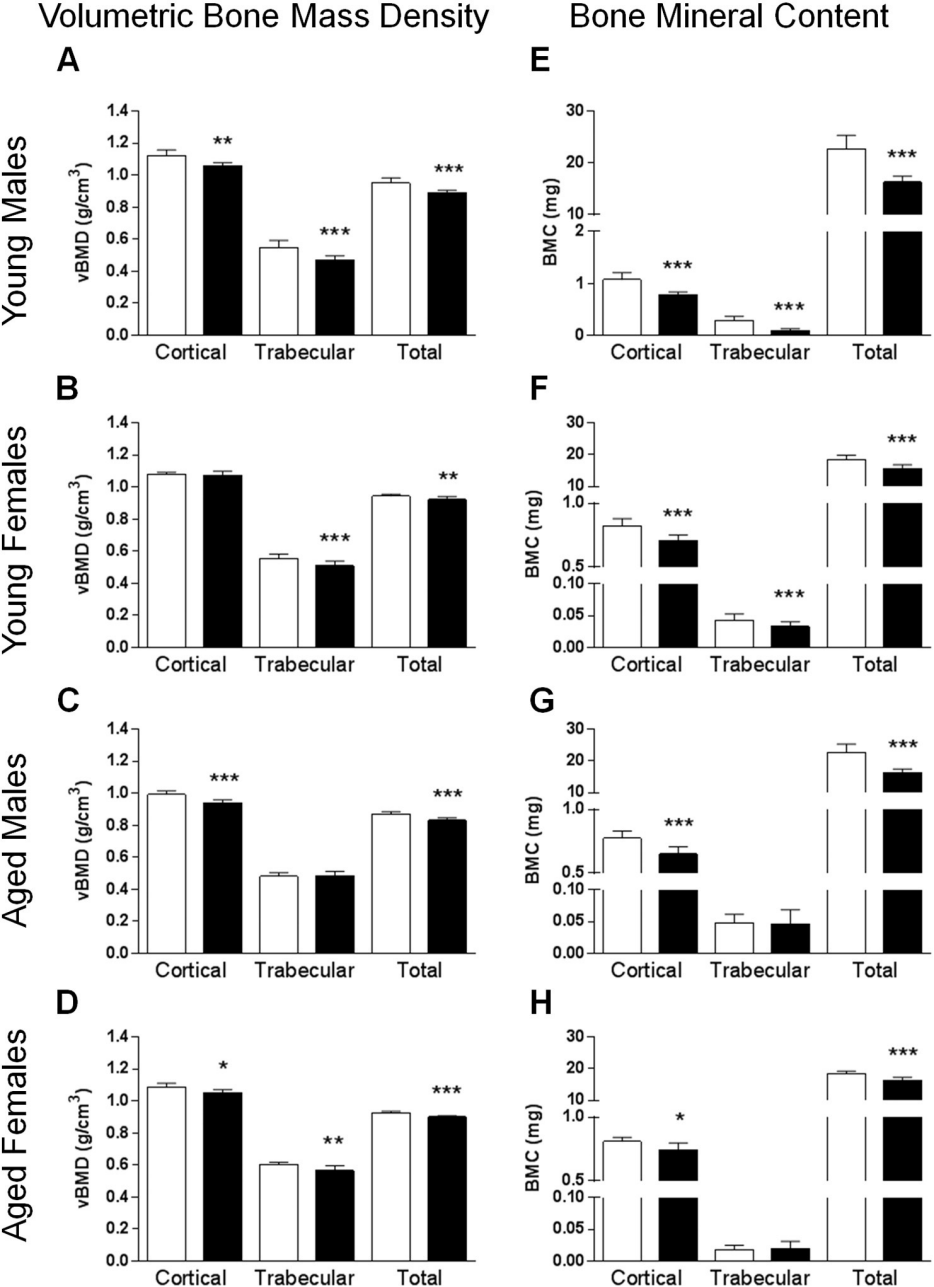
MR diet decreased vBMD and BMC in mice. Bone densitometry was analyzed by μCT in mice fed either CF (white bars) or MR (black bars) diets. Cortical, trabecular and total vBMD and BMC in young males (A and E, respectively), young females (B and F, respectively), aged males (C and G, respectively), and aged females (D and H, respectively). Statistical analysis was conducted using Student's unpaired *t*-test (*n* = 7–8/group, *P < 0.05, **P < 0.01, ***P < 0.001). vBMD, volumetric bone mineral density, BMC, bone mineral content CF, control-fed; MR, methionine restriction.

We next examined whether vBMD and BMC were affected by MR. Bone densitometry measurements indicated that cortical vBMD was lower in both groups of males and aged female MR mice than their CF counterparts (Fig. 2A, C, and D, *P* < 0.05), but not in young females (Fig. 2B). Trabecular vBMD was lower in young MR mice and aged female MR mice compared to their CF counterparts (Fig. 2A, B, and D, *P* < 0.01), but not in aged MR males (Fig. 2C). Total vBMD in all MR mice was lower compared to their respective CF counterparts (Fig. 2A–D, *P* < 0.01). In BMC, cortical and total BMC were lower in all MR mice than their CF counterparts (Fig. 2E–F, *P* < 0.05). Trabecular BMC was lower in young MR mice (Fig. 2E and F, *P* < 0.001), while aged CF and MR mice were similar to each other (Fig. 2G and H). When adjusted for body weight, cortical, trabecular and total vBMD were higher in both age groups of MR males compared to their CF counterparts (Table 1, Table 2, *P* < 0.001). Cortical and total vBMD were higher in young MR female while trabecular vBMD was not (Table 1, *P* < 0.01). No differences were observed in vBMD and BMC parameters in aged female mice (Table 2). BMC measurements in both age groups of MR males were higher compared to their CF counterparts (*P* < 0.05) while all BMC parameters remained the same in both age groups of female mice (Table 1, Table 2).

### 3.3 MR affected bone microarchitecture

We next tested whether MR affects the microarchitecture of mice as measured by micro-computed tomography (μCT). When adjusted for body weight, μCT measurements indicated that trabecular bone volume (BV), bone volume to trabecular volume ratio (BV/TV) and bone surface (BS) were lower (*P* < 0.05) while bone surface to trabecular volume ratio (BS/TV), structural model index (SMI), trabecular thickness (Tb.Th), trabecular space (Tb.Sp) were higher (*P* < 0.01) in young male MR mice compared to CF counterparts (Table 3). In addition, BS/TV in young females was higher in MR compared to CF (*P* < 0.01, Table 3). Aged MR males had lower BS and trabecular number (Tb.N) while BS/TV and SMI were higher compared to the CF group (P < 0.01), while all parameters in aged females were similar between CF and MR mice (Table 4). When the parameters were not adjusted for body weight, our data showed that MR reduced bone microarchitecture which was more pronounced in young males than young females and aged males while no effect was observed in aged females (Supplementary Tables 2 and 3). Representative images are depicted in Supplementary Fig. 2.

### 3.4 Intrinsic strength was preserved following MR diet

**Fig. 3:**
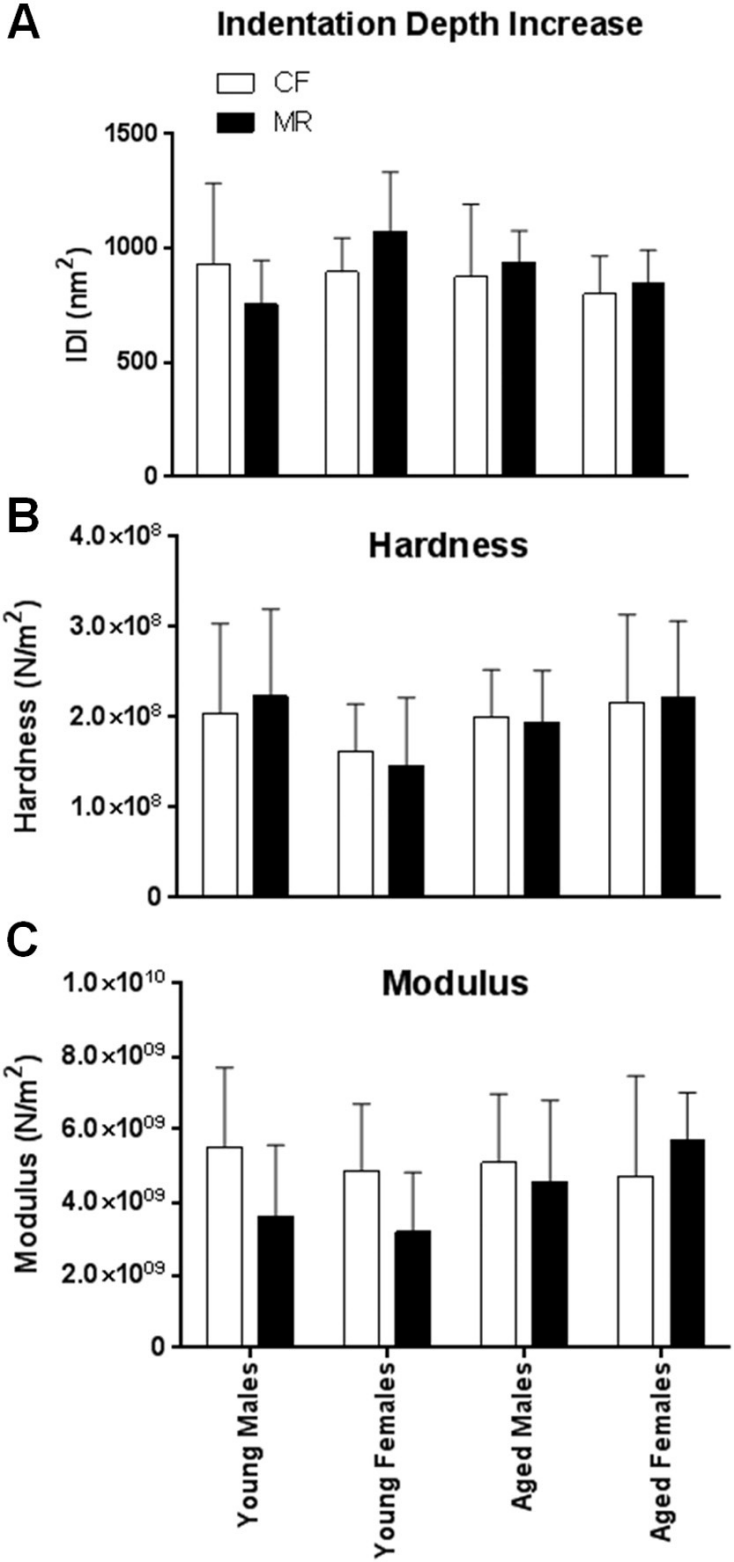
Intrinsic strength of bones was similar in CF and MR mice as revealed by nanoindentation tests. Indentation depth increase (A), hardness (B) and Modulus (C) were measured in the midshaft region of the femur, as described in the methods section in mice after 12 weeks of CF (white bars) or MR (black bars) diets. Statistical analysis was conducted between CF and MR of each age group and gender using Student's unpaired *t*-test (*n* = 5–8/group).

Our previous study in rats showed that MR reduced femoral extrinsic bending strength, but with increased intrinsic properties when compared to CF cohorts (Huang et al., 2014). In addition, after adjustment for body weight, femurs from ovariectomized (OVX) MR rats had higher stiffness compared to OVX CF cohorts (Huang et al., 1985). These studies suggest that the reduced bone strength of MR is size-related and could be independent of bone material properties. Thus, in the present study, we used nanoindentation tests to directly measure material properties of the bone at the tissue level without the influence of size, shape, and porosity (Rodriguez-Florez et al., 2013). Our data indicated that the indentation depth increase (IDI), hardness, and modulus were similar in young and aged male and female mice on CF and MR diets (Fig. 3A–C).

### 3.5 MR modified plasma hormone levels of mice

We next examined whether MR affected the hormones that are involved in bone metabolism as well as the hormones that are commonly affected by the diet (Table 5). We previously determined that the collagen synthesis marker P1NP and the collagen degradation marker CTX-1 were affected in HFD-MR mice (Ables et al., 2012). Our current data indicate that the P1NP levels were similar in young CF and MR mice and were elevated in aged MR mice compared to aged CF mice (*P* < 0.05). CTX-1 levels were elevated in young MR and aged male MR mice (*P* < 0.01), while the levels were similar in aged females.

Plasma RANKL levels were reduced in young and aged MR females (*P* < 0.05), while they remained similar in aged CF and MR males. Plasma OPG levels were reduced in MR males (*P* < 0.05), were elevated in young MR females (*P* < 0.05), and were similar in aged CF and MR females. The RANKL:OPG ratio was lower in young MR mice (male CF 8.1% ± 4.1% vs. male MR 4.6% ± 1.1%, *P* < 0.05; female CF 10% ± 3.9% vs. female MR 4.8% ± 2.3%, *P* < 0.05) and was similar in aged CF and MR mice (male CF 6.4% ± 4.4% vs. male MR 8.1% ± 2.4%; female CF 15% ± 4.7% vs. female MR 11% ± 3.4%).

We next assessed whether the hormones that are commonly affected by MR, such as leptin, IGF-1, adiponectin, and FGF21 (Miller et al., 2005, Ables et al., 2012, Ducy et al., 2000, Kajimura et al., 2013, Rosen et al., 2004, Wei et al., 2012), were regulated in our cohorts. Our data indicate that leptin levels were reduced in MR males (*P* < 0.01), but remained similar in CF and MR females. Additionally, IGF-1 was reduced (*P* < 0.01), while adiponectin and FGF21 were elevated (*P* < 0.05 for both hormones) in MR mice compared to CF mice which was consistent with our previous data (Miller et al., 2005, Ables et al., 2012).

### 3.6 MR downregulated collagen formation and bone differentiation genes

**Fig. 4:**
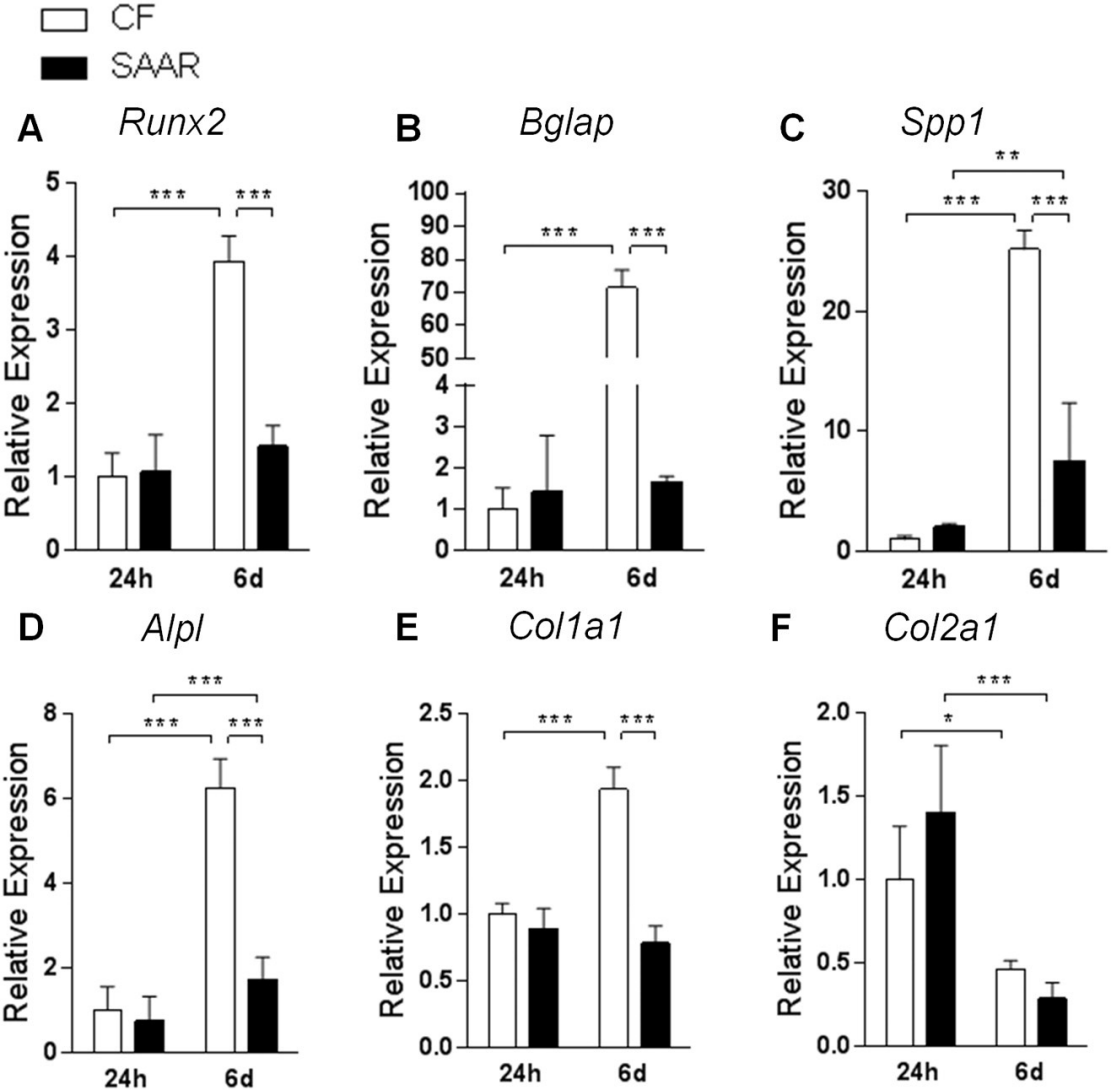
Sulfur amino acid restriction (SAAR) downregulated collagen formation and bone differentiation genes in MC3T3-E1 cells. Mouse preosteoblast cell line, MC3T3-E1 subclone 4 were cultured in either complete media (CF, white bars) or SAAR (black bars) media containing 80% reduced cysteine, cystine and methionine concentrations as described in the method section. TaqMan gene expression analyses for (A) *Runx2*, (B) *Bglap*, (C) *Spp1*, (D) *Alpl*, (E) *Col1a1* and (F) *Col2a1* at 24 h and after 6 days incubation. Statistical analysis was conducted using 2-way ANOVA of both time points between CF and MR (*n* = 6/group).

To gain insight on the possible molecular mechanism of MR in bones we used mouse preosteoblast cell line, MC3T3-E1 subclone 4 as described previously (Quarles et al., 1992, Franceschi et al., 1994). Gene expression markers for collagen formation and bone differentiation remained similar between CF and SAAR treated cells after 24 h in culture (Fig. 4). After 6 days in culture, the molecular signature of CF treated cells suggests osteoblast differentiation as indicated by the upregulation of *Runx2*, *Bglap*, *Spp1*, *Alpl* and *Col1a1* (Fig. 4A–E, *P* < 0.001) genes when compared to 24 h CF cells. In contrast, SAAR attenuated differentiation by the cells as indicated by the similar gene expression levels of *Runx2*, *Bglap* and *Col1al* (Fig. 4A, B, and E). *Spp1* and *Alpl* were upregulated in SAAR treated cells after 6 days, but did not reach CF levels of expression (Fig. 4C and D, *P* < 0.01). Chondrogenic marker *Col2a1* was similar in CF- and SAAR-treated cells in both time points and was downregulated after 6 days of differentiation when compared to 24 h (Fig. 4F, *P* < 0.05).

To test whether the genes that were affected by MR in cells were also altered in whole bones, we isolated RNA from femurs of aged males and females (Supplementary Fig. 3). Gene expression analysis indicated that *Bglap* was downregulated in aged MR males compared to its CF counterpart (Supplementary Fig. 3A). All other genes tested were not affected by the diet in aged female mice (Supplementary Fig. 3B).

## 4 Discussion

The aim of the study was to evaluate the effect of MR on bones in young and aged male and female C57BL/6J mice. Our results indicate that MR reduces bone mass and alters the bone microarchitecture while preserving the intrinsic strength of mice. When adjusted for body weight, our data indicate that the lower bone mass in MR mice is appropriate for its smaller body size. Our present work extends previous studies on MR mice on HFDs (Ables et al., 2012) and on rats during exercise (Huang et al., 2014), both of which exhibited reduced bone mass. In this study, we show variable age- and gender-specific effects of MR on bones in mice. Finally, we identified the possible molecular mechanism in a mouse preosteoblast cell line, which showed attenuated levels of gene expression for collagen formation and bone differentiation markers.

The present work indicates that the effects of MR on bone were comparable to studies conducted using a low-protein diet (LPD) in rodents (Ammann et al., 2000, Bozzini et al., 2011). Previous studies that characterized the effects of LPD in rodent bones reduced casein by 83%, which decreased body weight, bone formation, and bone mass (Dubois-Ferriere et al., 2014, Bourrin et al., 2000a, Bourrin et al., 2000b) without affecting extrinsic bone strength (Dubois-Ferriere et al., 2014). Other studies manipulated the protein quality (e.g., casein, gluten, or soy) or concentration and characterized their effects on bones (Alippi et al., 2012, Rouy et al., 2014). Gluten or a low percentage of soy protein negatively affected the bone cross-sectional geometry and structural properties in growing female rats (Alippi et al., 2012). However, when normalized to body weight, the differences in strength and stiffness disappeared (Alippi et al., 2012). In addition, a low-soy diet diminished the femoral cortical thickness, BV, and Tb.N, and Tb.Th and increased medullar adiposity in growing mice, but it did not affect the trabecular bone after correcting for body weight (Rouy et al., 2014). Taken together, these studies suggest that the effects of LPDs in bones may be due to a concomitant subnormal gain in body weight. Our data agree with these conclusions, as the observed reductions in the bone parameters disappeared or were increased in MR mice despite their small stature.

An important characteristic of MR was its effects on the properties of the bone material level. Bozzini et al. suggested that growing female rats on a LPD reduced the mandibular bone structural properties as a consequence of a correlative loss of gain in both growth and mass, but not in the bone material properties (Bozzini et al., 2011). Additionally, Allipi, et al. demonstrated that bone material quality indicators (elastic modulus, yielding stress, elastic energy absorption/volume) were not affected by LPDs (Alippi et al., 2012). Furthermore, indirect calculations showed that MR rats had higher intrinsic biomechanical strength and toughness compared to CF rats, suggesting that despite their smaller stature, MR may be beneficial to bone material properties (Huang et al., 2014). Finally, ovariectomized MR rats exhibited larger and stronger biomechanical properties compared to CF counterparts when adjusted for body weight (Huang et al., 1985). Overall, our data supports previous studies (Huang et al., 2014, 1985) that showed MR compromised bone mass, bone size and/or whole bone strength and verified that, indeed, the diet does not affect bone material properties.

Bone development is a summary of modeling/remodeling activities, which are influenced by hormones that are secreted either by cells within the tissue or in an endocrine manner. The effects of MR on the hormones involved in bone remodeling were diverse in our studies. Our data on P1NP in young mice are consistent with HFD-MR mice, where the levels were similar in both the CF and MR groups (Ables et al., 2012). Interestingly, the P1NP levels were higher in aged male MR mice than aged male CF mice, suggesting more active bone formation. Consistent with our previous studies, our current data show that male and young female MR mice had elevated CTX-1 levels, suggesting that the reduced bone mass could be partially due to increased collagen degradation (Ables et al., 2012). However, male MR rats had lower CTX-1 levels compared to their CF counterparts, demonstrating species differences in the hormonal regulation of CTX-1 under MR conditions (Huang et al., 2014). Therefore, the elevated levels of both P1NP and CTX-1 suggest active bone remodeling in MR mice, which could explain the low bone mass phenotype. In contrast to bone specific markers, serum RANKL and OPG, which are indirect indices for bone turnover, did not suggest activated osteoclastogenesis. Because RANKL and OPG are also expressed by multiple cell types (e.g., B cells and T cells), which are mediated by immune as well as inflammatory responses (Kearns et al., 2008), the down-regulation of RANKL and OPG was more likely the result of a collaboration with the multi-regulated physiological system rather than acting as a specific indicator for bone cell activities. This is supported by data on the hormones that were affected by MR, such as IGF-1, the adipokine leptin and adiponectin, and the hepatokine FGF21. Our data is consistent with previous reports in which MR decreased IGF-1 levels in rodents (Ables et al., 2012, Huang et al., 2014, Malloy et al., 2006, Perrone et al., 2010), MR had less impact on bone mass and structure in aged female mice, suggesting age- and gender-specific alternate pathways of IGF-1 in the bones of MR mice. Leptin has been shown to increase the osteoblast number and activity (Turner et al., 2013). Since MR reduced leptin levels in males, but not in females, could partly explain the low bone mass phenotype (Ducy et al., 2000, Turner et al., 2013). Adiponectin decreases bone mass by inhibiting the proliferation of osteoblasts and reducing osteocalcin levels (Kajimura et al., 2013). However, chronic elevated adiponectin levels increase bone mass via neuronal activation of the PI3-kinase-FoxO1 pathway (Kajimura et al., 2013). MR consistently led to elevated levels of adiponectin in mice, but had varying effects on bones, suggesting its age- and gender-specific influence on bone metabolism. Elevated FGF21 levels reduce bone mass by inhibiting osteoblastogenesis and increasing bone marrow adipogenesis via increased peroxisome-proliferator activator-receptor-gamma activity (Wei et al., 2012). Our data indicate that elevated FGF21 levels in MR mice could induce low bone mass, at least in young MR and aged MR males, but not in aged MR females, suggesting an age- and gender-specific effect of the diet on development. Collectively, the effects of MR on bones are regulated by a collaboration of hormones that are secreted by bone tissue and peripheral organs.

A potential consequence of hormonal regulation on bone remodeling was observed in the changes in the vBMD, bone size, and body mass of the study groups. In our previous study in MR rats, cross-sectional measurements of long bones and BMC indices were consistently correlated with body weight, suggesting that smaller bones are most likely the result of a body size-related phenomenon (Huang et al., 2014). In addition, our current data indicate that when normalized for body weight, total vBMD and BMC were higher in male MR mice. Because the body weight of aged females was unchanged for the duration of the study, it served as a negative control for our experiments. Therefore, under the current MR conditions, the animals demonstrated different levels of body size as well as bone mass change without further compromising bone material properties.

Of particular interest, female mice were affected by MR less than male mice. The use of retired breeder female mice as the aged female group served as a model for postmenopausal women. Our data on aged female mice show that body weight and bone microarchitecture were similar in CF and MR mice. One possible explanation could be the interference of estrogen, which is also involved in bone remodeling (Connelly et al., 2015, Henning et al., 2014, Macari et al., 2015). A study on pregnant women showed high bone turnover which could explain trabecular bone loss (Naylor et al., 2000). In addition, because the dependence on methionine decreased with aging (Pitkanen et al., 2003), MR had the lowest impact on the body size and bones of aged female mice, suggesting that the dosage of MR in aged female mice used in this study was not optimal. Therefore, it is recommended that appropriate and carefully titrated dosages of MR in aged females be conducted.

Studies using 40% MR in juvenile rats did not affect growth as represented by body weight (Sanchez-Roman and Barja, 2013) but nonetheless reduced oxidative stress markers (Caro et al., 2008, Sanchez-Roman et al., 2011) when compared to control counterparts. The possible mechanism is the similar levels of reductions in mtROSp and 8-oxodG in mtDNA in both MR conditions (Caro et al., 2009). Therefore, future studies could be directed in determining the effects of 40% MR on bones.

To our knowledge, this is the first study that used mouse preosteoblast cell line MC3T3-E1 subclone 4 under low sulfur amino acid conditions to identify the molecular mechanism of MR. MC3T3-E1 cells have been used extensively to characterize and determine molecular mechanisms of osteoporosis in mouse bone cells (Xiao et al., 1997, Franceschi et al., 1994, Addison et al., 2015). Additionally, SAAR in cell growth media was established in renal cell line studies on epithelial tight junctions (Ramalingam et al., 2010, Skrovanek et al., 2007). These studies enabled us to determine the potential molecular mechanism of MR at the cellular level. In agreement with previous studies, CF-treated cells showed upregulation of *Runx2*, *Bglap*, *Spp1*, *Alpl* and *Col1a1* gene expression markers for osteoblast differentiation (Wang et al., 1999, Beck et al., 1998, Roca et al., 2005). However, expression for the same differentiation genes in SAAR-treated cells remained similar even after 6 days of culture suggesting its repressed differentiation. In addition, chondrogenic marker *Col2a1* (Oralova et al., 2015) was downregulated in both treatment groups after 6 days. Based on our results, the attenuation of gene expression markers for collagen formation and bone differentiation in MC3T3-E1 cells under SAAR conditions suggests delays in bone differentiation and could possibly explain the reduced bone parameters observed in mice. The effect of low methionine on bone differentiation was further confirmed when *Bglap* was downregulated in whole bones of aged male MR mice. Overall, the possible molecular mechanism of MR could be due to its effects on genes that affect bone differentiation.

In conclusion, our study establishes the following effects of MR on bones: 1) bone morphology in mice is altered, possibly under hormonal regulation in an age- and gender-specific manner, 2) reduced bone mass in MR mice is relative and appropriate for its body size and does not impair the material-level biomechanical properties and 3) attenuated gene expression levels for bone differentiation and collagen synthesis in vitro could cause, at least in part, delays in bone formation and overall growth. Overall, our studies extend the current knowledge of MR and augment the parameters necessary for clinical trials.

## Author contributions

AO, DC, RG and TH and GA made substantial contributions to acquisition and analysis data; TH and GA drafted the manuscript and revised it critically for important intellectual content; AO, DC, RG, TH and GA approved the final version of the submitted manuscript.

## Funding

Orentreich Foundation for the Advancement of Science, Inc (ASL12).

## Figures and Tables

**Table 1:** Body-weight adjusted vBMD and BMC bones in young mice following CF and MR diets for 12 weeks. Comparisons between same sex CF and MR were conducted using Student's unpaired *t*-test (*n* = 7–8/group).

	Young males		Young females	
	CF	MR	CF	MR
vBMD (mg/cm^3^) to BW (g)				
Cortical	34.4 ± 1.7	50.2 ± 2.9⁎⁎⁎	48.9 ± 3.4	55.6 ± 2.9⁎⁎
Trabecular	16.8 ± 1.4	22.3 ± 1.1⁎⁎⁎	25.2 ± 2.7	26.4 ± 1.7
Total	29.2 ± 1.3	42.1 ± 2.4⁎⁎⁎	42.7 ± 2.7	47.7 ± 2.5⁎⁎
BMC (μg) to BW (g)				
Cortical	32.8 ± 3.3	36.8 ± 2.7⁎	37.1 ± 1.6	36.6 ± 2.3
Trabecular	8.7 ± 2.5	4.5 ± 1.1⁎⁎	1.9 ± 0.5	1.7 ± 0.4
Total	696 ± 68	770 ± 47⁎	834 ± 28	812 ± 43

⁎ *P* < 0.05.

⁎⁎ *P* < 0.01.

⁎⁎⁎ *P* < 0.001.

**Table 2:** Body-weight adjusted vBMD and BMC bone in aged mice following CF and MR diets for 12 weeks. Comparisons between same sex CF and MR were conducted using Student's unpaired *t*-test (*n* = 7–8/group).

	Aged males		Aged females	
	CF	MR	CF	MR
vBMD (mg/cm^3^) to BW (g)				
Cortical	25 ± 1.9⁎	34.2 ± 3.1⁎⁎⁎	41.5 ± 2.6	43 ± 1.7
Trabecular	12.1 ± 0.9⁎⁎	17.6 ± 1.6⁎⁎⁎	23.1 ± 1.2	23.1 ± 0.7
Total	21.9 ± 1.4	30.3 ± 2.9⁎⁎⁎	35.4 ± 2	36.8 ± 1.2
BMC (μg) to BW (g)				
Cortical	19.5 ± 1.8	23.5 ± 1.3⁎⁎⁎	31 ± 2.5	30.3 ± 1.6
Trabecular	1.2 ± 0.3	1.6 ± 0.6	0.7 ± 0.3	0.8 ± 0.5
Total	435 ± 28	527 ± 35⁎⁎⁎	703 ± 53	663 ± 36

⁎ *P* < 0.05.

⁎⁎ *P* < 0.01.

⁎⁎⁎ *P* < 0.001.

**Table 3:** Body-weight adjusted trabecular and midshaft bone microarchitecture in young mice following CF and MR diets for 12 weeks. Comparisons between same sex CF and MR were conducted using Student's unpaired *t*-test (*n* = 7–8/group). BV, bone volume; TV, total volume; BS, bone surface; SMI, structure model index; Tb.Th, trabecular thickness; Tb.N trabecular number; Tb.Sp, trabecular separation; Conn.Dn., connectivity density; MOI, moment of inertia; pMOI, polar MOI; *I*_*max*_, maximal MOI; *I*_*min*_, minimal MOI.

	Young males		Young females	
	CF	MR	CF	MR
Trabecular bone				
BV (mm^3^)	0.016 ± 0.004	0.009 ± 0.002⁎⁎	0.003 ± 0.001	0.003 ± 0.001
BV/TV (%)	0.561 ± 0.12	0.382 ± 0.05⁎⁎	0.176 ± 0.04	0.176 ± 0.04
BS (mm^2^)	0.721 ± 0.1	0.601 ± 0.09⁎	0.221 ± 0.05	0.229 ± 0.03
BS/TV (1/mm)	1.49 ± 0.33	3.09 ± 0.39⁎⁎⁎	2.94 ± 0.25	3.68 ± 0.45⁎⁎
SMI	0.073 ± 0.01	0.128 ± 0.01⁎⁎	0.142 ± 0.01	0.153 ± 0.01
Tb.Th (mm)	0.0025 ± 0.0	0.0029 ± 0.0⁎⁎⁎	0.003 ± 0.0	0.003 ± 0.0
Tb.N (1/mm)	0.068 ± 0.01	0.06 ± 0.01	0.024 ± 0.004	0.028 ± 0.006
Tb·Sp. (mm)	0.008 ± 0.002	0.014 ± 0.001⁎⁎⁎	0.021 ± 0.003	0.022 ± 0.003
Conn.Dn. (1/mm^3^)	3.3 ± 0.52	3.0 ± 0.43	3.9 ± 2.1	2.8 ± 1.4
Midshaft tibia				
Bone area (mm^2^)	0.029 ± 0.003	0.034 ± 0.002⁎⁎	0.034 ± 0.002	0.033 ± 0.002
pMOI (mm^4^)	0.009 ± 0.002	0.01 ± 0.001	0.009 ± 0.001	0.008 ± 0.001
*I*_*max*_ (mm^4^)	0.005 ± 0.001	0.006 ± 0.001	0.005 ± 0.00	0.004 ± 0.00
*I*_*min*_ (mm^4^)	0.003 ± 0.001	0.004 ± 0.001	0.004 ± 0.00	0.003 ± 0.00

⁎ *P* < 0.05.

⁎⁎ *P* < 0.01.

⁎⁎⁎ *P* < 0.001.

**Table 4:** Body-weight adjusted trabecular and midshaft bone microarchitecture in aged mice following CF and MR diets for 12 weeks. Comparisons between same sex CF and MR were conducted using Student's unpaired *t*-test (*n* = 7–8/group). BV, bone volume; TV, total volume; BS, bone surface; SMI, structure model index; Tb.Th, trabecular thickness; Tb.N trabecular number; Tb.Sp, trabecular separation; Conn.Dn., connectivity density; MOI, moment of inertia; pMOI, polar MOI; *I*_*max*_, maximal MOI; *I*_*min*_, minimal MOI.

	Aged males		Aged females	
	CF	MR	CF	MR
Trabecular bone				
BV (mm^3^)	0.002 ± 0.001	0.002 ± 0.001	0.001 ± 0.00	0.001 ± 0.001
BV/TV (%)	0.102 ± 0.03	0.09 ± 0.03⁎	0.053 ± 0.02	0.06 ± 0.03
BS (mm^2^)	0.145 ± 0.03	0.138 ± 0.04⁎⁎	0.065 ± 0.03	0.069 ± 0.03
BS/TV (1/mm)	1.6 ± 0.18	2.49 ± 0.42⁎⁎⁎	2.2 ± 0.26	2.35 ± 0.23
SMI	0.078 ± 0.01	0.117 ± 0.01⁎⁎⁎	0.113 ± 0.01	0.118 ± 0.008
Tb.Th (mm)	0.002 ± 0.0	0.003 ± 0.0	0.003 ± 0.0	0.003 ± 0.0
Tb.N (1/mm)	0.015 ± 0.004	0.013 ± 0.003⁎⁎⁎	0.007 ± 0.003	0.007 ± 0.004
Tb.Sp (mm)	0.009 ± 0.001	0.016 ± 0.002	0.027 ± 0.002	0.029 ± 0.002
Conn.Dn. (1/mm^3^)	0.83 ± 0.3	0.75 ± 0.32	0.78 ± 0.41	0.43 ± 0.28
Midshaft tibia				
Bone area (mm^2^)	0.019 ± 0.002	0.025 ± 0.002	0.029 ± 0.002	0.029 ± 0.002
pMOI (mm^4^)	0.006 ± 0.001	0.008 ± 0.001	0.008 ± 0.001	0.008 ± 0.001
*I*_*max*_ (mm^4^)	0.004 ± 0.00	0.005 ± 0.00	0.004 ± 0.00	0.005 ± 0.00
*I*_*min*_ (mm^4^)	0.002 ± 0.00	0.003 ± 0.00	0.004 ± 0.00	0.004 ± 0.00

⁎ *P* < 0.05.

⁎⁎ *P* < 0.01.

⁎⁎⁎ *P* < 0.001.

**Table 5:** Plasma hormone levels of young male and female mice on CF and MR diets for 12 weeks. Comparisons between same sex CF and MR were conducted using Student's unpaired *t*-test (*n* = 7–8/group). P1NP, N-terminal propeptide of type 1 procollagen; CTX-1, C-terminal telopeptide of type 1 collagen; RANKL, receptor activator for nuclear factor κB ligand; IGF-1, insulin-like growth factor-1; FGF21, fibroblast growth factor-21; OPG, osteoprotegerin; OC, osteocalcin.

	Young males		Young females		Aged males		Aged females	
	CF	MR	CF	MR	CF	MR	CF	MR
P1NP (ng/ml)	40 ± 8	39 ± 9	42 ± 11	49 ± 15	34 ± 3	44 ± 7⁎⁎	32 ± 8	41 ± 3⁎
CTX-1 (ng/ml)	16 ± 1	25 ± 7⁎⁎	21 ± 5	33 ± 7⁎⁎	14 ± 2	20 ± 3⁎⁎	18 ± 4	20 ± 5
RANKL (pg/ml)	142 ± 66	77 ± 13⁎	159 ± 41	100 ± 30⁎	122 ± 31	122 ± 50	223 ± 40	179 ± 32⁎
OPG (ng/ml)	1.9 ± 0.6	1.6 ± 0.2	1.7 ± 0.4	2.2 ± 0.4⁎	2.1 ± 0.6	1.6 ± 0.4⁎	1.6 ± 0.3	1.7 ± 0.4
OC (ng/ml)	31 ± 5	27 ± 2	33 ± 6	39 ± 7	26 ± 3	28 ± 4	25 ± 2	27 ± 4
Leptin (ng/ml)	11 ± 8	0.9 ± 0.3⁎⁎	1.6 ± 0.7	1.1 ± 0.5	32 ± 14	3.4 ± 1.3⁎⁎⁎	1.6 ± 0.5	1.3 ± 0.6
IGF-1 (pg/ml)	355 ± 60	182 ± 42⁎⁎⁎	366 ± 99	209 ± 47⁎⁎	674 ± 202	277 ± 80⁎⁎⁎	414 ± 80	276 ± 32⁎⁎
Adiponectin (μg/ml)	7.7 ± 1.9	15.7 ± 0.9⁎⁎⁎	14.8 ± 1.3	18.9 ± 1.0⁎⁎⁎	9.8 ± 1.5	11.8 ± 1.7⁎	11.3 ± 1.1	14.9 ± 1.9⁎⁎
FGF21 (ng/ml)	0.82 ± 0.5	6.0 ± 2.1⁎⁎	0.44 ± 0.2	3.8 ± 1.1⁎⁎⁎	0.66 ± 0.5	1.6 ± 0.6⁎	0.9 ± 0.3	2.6 ± 0.9⁎⁎

⁎ *P* < 0.05.

⁎⁎ *P* < 0.01.

⁎⁎⁎ *P* < 0.001.
