# Supplementary material for: Methionine restriction alters bone morphology and affects osteoblast differentiation
Source: Bone Rep. 2016 Feb 11;5:33–42. doi: 10.1016/j.bonr.2016.02.002 (PMC4926829; doi:10.1016/j.bonr.2016.02.002)
Supplement: Supplementary file 1 — Supplementary material [file mmc1.pdf]

Methionine restriction alters bone morphology and affects osteoblast differentiation

Amadou Ouattara[1], Diana Cooke[1], Raj Gopalakrishnan[2], Tsang-hai Huang[3],and Gene P. Ables[1*]

[1]The Orentreich Foundation for the Advancement of Science, Cold Spring, NY 10516, [2]School of Dentistry, University of Minnesota, Minneapolis, MN 55455, [3]Institute of Physical Education, Health and Leisure Studies, National Cheng Kung University, Tainan, Taiwan

Supplementary Table 1. Formula of MR (CF) diets used in the study.

Ingredients	g / 100 g
L-Arginine	1.09
L-Histidine	0.32
L-Isoleucine	0.80
L-Leucine	1.08
L-Lysine	1.40
DL-Methionine	0.12 (0.84)
L-Phenylalanine	1.13
L-Threonine	0.80
L-Tryptophan	0.17
L-Valine	0.80
L-Glutamic Acid	3.34 (2.62)
Glycine	2.26
Corn Starch	53.38
Dextrose	4.86
Sucrose	14.57
Cellulose	4.86
Corn Oil	4.47
Minerals	3.40
Vitamins	0.97
Choline Bitartrate	0.19
Total	100.00

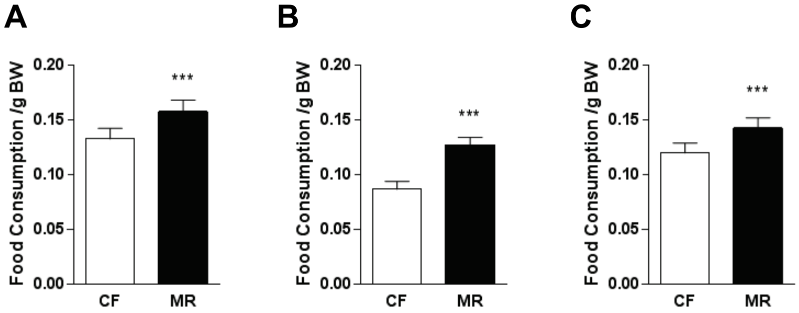

Supplementary Figure 1. Food consumption per gram body weight of young female (A) and aged male and female (B and C) mice on CF (white bars) and MR (black bars) diets for 12 weeks. Statistical analysis was conducted using Student's unpaired *t*-test (n = 7 – 8 / group, ***P < 0.001).

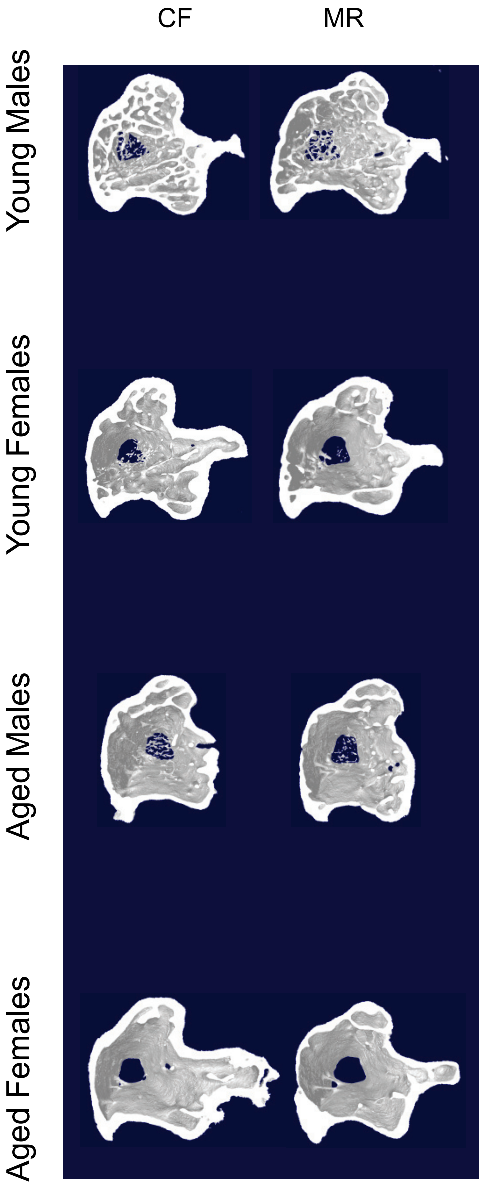

Supplementary Figure 2. Representative µCT slices from tibiae of young and aged male and female mice on CF and MR diets for 12 weeks. µCT: microcomputed tomography.

Supplementary Table 2. Trabecular and midshaft bone microarchitecture in young mice following CF and MR diets for 12 weeks. Comparisons between same sex CF and MR were conducted using Student's unpaired *t*-test (n = 7 – 8 / group, $^{*}P < 0.05$, $^{**}P < 0.01$, $^{***}P < 0.001$).

	Young Males		Young Females	
	CF	MR	CF	MR
Trabecular Bone				
BV (mm^3)	0.51 ± 0.13	0. 0.20 ±05***	0.08 ± 0.02	0.06 ± 0.01
BV/TV (%)	18.3 ± 4.1	8.12 ± 1.4***	3.88 ± 0.8	3.38 ± 0.7
BS (mm^2)	23.5 ± 3.6	12.7 ± 2.2***	4.88 ± 0.9	4.42 ± 0.6
BS/TV (1/mm)	48.0 ± 8.7	65.0 ± 5.9***	65.1 ± 5.0	70.8 ± 7.2
SMI	2.36 ± 0.2	2.69 ± 0.1**	3.13 ± 0.1	2.95 ± 0.2*
Tb.Th. (mm)	0.08 ± 0.01	0.06 ± 0.01***	0.07 ± 0.01	0.06 ± 0.01*
Tb.N. (1/mm)	2.20 ± 0.36	1.28 ± 0.14***	0.53 ± 0.09	0.53 ± 0.1
Tb.Sp. (mm)	0.25 ± 0.04	0.29 ± 0.04	0.45 ± 0.06	0.43 ± 0.05
Conn.Dn (1/mm^3)	108 ± 19	64.4 ± 10.6***	84.1 ± 40.9	53.1 ± 26.5
Midshaft Tibia				
Bone area (mm^2)	0.94 ± 0.12	0.73 ± 0.05***	0.74 ± 0.05	0.65 ± 0.04***
pMOI (mm^4)	0.29 ± 0.07	0.21 ± 0.03**	0.19 ± 0.03	0.15 ± 0.02*
I_{max} (mm^4)	0.18 ± 0.05	0.13 ± 0.02*	0.10 ± 0.02	0.09 ± 0.01*
I_{min} (mm^4)	0.11 ± 0.02	0.08 ± 0.01*	0.09 ± 0.01	0.07 ± 0.01

Supplementary Table 3. Trabecular and midshaft bone microarchitecture in aged mice following CF and MR diets for 12 weeks. Comparisons between same sex CF and MR were conducted using Student's unpaired *t*-test (n = 7 – 8 / group, $^{*}P < 0.05$, $^{**}P < 0.01$, $^{***}P < 0.001$).

	Aged Males		Aged Females	
	CF	MR	CF	MR
Trabecular Bone				
BV (mm^3)	0.09 ± 0.03	0.06 ± 0.02*	0.03 ± 0.01	0.03 ± 0.02
BV/TV (%)	4.03 ± 1.1	2.43 ± 0.8**	1.39 ± 0.6	1.35 ± 0.7
BS (mm^2)	5.77 ± 1.2	3.79 ± 1.3**	1.68 ± 0.6	1.68 ± 0.8
BS/TV (1/mm)	63.7 ± 5.2	66.8 ± 5.5	58.6 ± 5.7	57.4 ± 4.3
SMI	3.09 ± 0.2	3.16 ± 0.2	2.97 ± 0.2	2.88 ± 0.2
Tb.Th. (mm)	0.07 ± 0.00	0.07 ± 0.00	0.08 ± 0.01	0.08 ± 0.01
Tb.N. (1/mm)	0.58 ± 0.2	0.35 ± 0.1**	0.18 ± 0.1	0.17 ± 0.1
Tb.Sp. (mm)	0.37 ± 0.04	0.43 ± 0.03**	0.71 ± 0.04	0.72 ± 0.06
Conn.Dn (1/mm^3)	33.0 ± 11.1	20.2 ± 7.64*	20.7 ± 11.4	10.7 ± 6.81
Midshaft Tibia				
Bone area (mm^2)	0.77 ± 0.05	0.69 ± 0.05**	0.75 ± 0.02	0.70 ± 0.05
pMOI (mm^4)	0.23 ± 0.03	0.21 ± 0.04	0.20 ± 0.02	0.21 ± 0.03
I_{max} (mm^4)	0.14 ± 0.02	0.13 ± 0.03	0.11 ± 0.01	0.12 ± 0.02
I_{min} (mm^4)	0.09 ± 0.01	0.08 ± 0.01	0.09 ± 0.01	0.09 ± 0.01

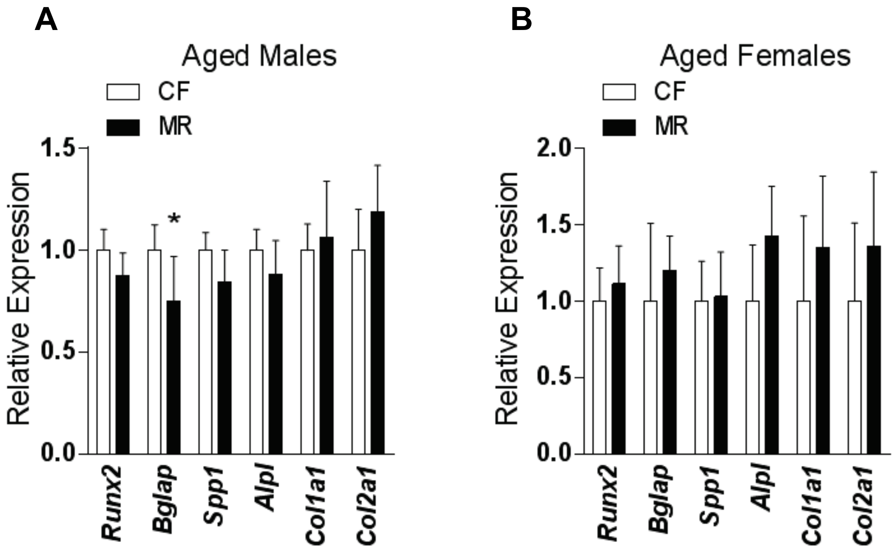

Supplementary Fig 3. Whole bone gene expression analyses from aged males and females. Whole bone RNA was isolated from femurs of aged males (A) and aged females (B) as described in the methods section. TaqMan gene expression analyses was done using primers for *Runx2*, *Bglap*, *Spp1*, *Alpl*, *Col1a1* and *Col2a1*. Statistical analysis was conducted using Student's unpaired *t*-test between CF and MR (n = 6 - 8 / group, $^{*}P < 0.05$).
